# *Bulletin* comment: Ever-expanding empires^†^

**DOI:** 10.1192/pb.bp.114.047837

**Published:** 2015-04

The development of policy and governmental decisions at the highest level ought to be based on sound information, research or expert advice. Further, once change has been implemented and new practices set up there needs to be ongoing assessment, monitoring and reflection on the relative merits of the situation. Depending on the findings it might be necessary to make further changes: sometimes these alterations may even result in a complete reversal of the newly implemented practice.

The plan to increase the availability of psychological therapy for individuals with the more common mental disorders was clearly a good one. However, in practice the evidence for its effectiveness failed to materialise; research originating from two pilot sites was flawed giving an unbelievable impression of it doing good. Hopes of returning thousands of people with mental health issues to full-time employment were also unmet. Incredibly, the response to such an unconvincing success has been the further expansion of the service into the provision of talking treatments for children and young people experiencing a wide range of psychological problems.

No one can argue against the expansion of psychological services so that much needed appropriate treatment can be given to people with genuine problems. However, any plan consuming millions of public money ought to show convincing effectiveness on all fronts before it is blindly expanded.

